# Reinforcement of patient education improved physicians’ adherence to guideline-recommended medical therapy after acute coronary syndrome

**DOI:** 10.1371/journal.pone.0217444

**Published:** 2019-06-06

**Authors:** Chih-Kuo Lee, Chao-Lun Lai, Ming-Hsien Lee, Fang-Ying Su, Tzu-Shan Yeh, Li-Ying Cheng, Mu-Yang Hsieh, Yen-Wen Wu, Yen-Bin Liu, Chih-Cheng Wu

**Affiliations:** 1 Department of Internal Medicine, National Taiwan University Hospital Hsin-Chu Branch, Hsinchu, Taiwan; 2 Department of Internal Medicine, College of Medicine, National Taiwan University, Taipei, Taiwan; 3 Institute of Epidemiology and Preventive Medicine, College of Public Health, National Taiwan University, Taipei, Taiwan; 4 Department of Nursing, National Taiwan University Hospital, Hsin-Chu Branch, Hsinchu, Taiwan; 5 Biotechology R&D Center, National Taiwan University Hospital Hsin-Chu Branch, Hsinchu, Taiwan; 6 Division of Cardiology, Cardiovascular Medical Center, and Department of Nuclear Medicine, Far Eastern Memorial Hospital, New Taipei City, Taiwan; 7 National Yang-Ming University School of Medicine, Taipei, Taiwan; 8 Cardiovascular Center, National Taiwan University Hospital Hsin-Chu Branch, Hsinchu, Taiwan; 9 Institute of Biomedical Engineering, National Tsing-Hwa University, Hsinchu, Taiwan; East Tennessee State University, UNITED STATES

## Abstract

**Background:**

Prescription of guideline-recommended medicines after acute coronary syndrome (ACS) has been suboptimal. Tools for improving the use of medications have been developed, but they mainly targeted physicians.

**Objective:**

We evaluated the effects of reinforcement of patient and family education on the usage of guideline-recommended secondary prevention medications.

**Methods:**

This was a retrospective analysis of a prospectively collected registry of patients with ACS who were admitted to a regional teaching hospital in Taiwan between February 2015 and April 2017. The control group included 76 patients discharged before implementing the electronic-based patient and family education (PFE) system. The intervention group included 206 patients discharged after implementation. The primary outcome was the prescription rate of all four guideline-recommended drugs. Predictors of adherence were also evaluated.

**Results:**

The study cohort included 282 ACS patients (188 men and 94 women) with a mean age of 68.5 years (standard deviation, 14.2). The intervention group patients were younger, had more family history of premature cardiovascular disease, more dyslipidemia, and underwent more reperfusion therapy. The intervention group was prescribed more guideline-recommended drugs than the control group: dual antiplatelet agents, 79.61% vs. 47.37% (p<0.001); statins, 74.76% vs. 34.21% (p<0.001); beta-blockers, 81.07% vs. 46.05% (p<0.001); angiotensin-converting enzyme inhibitors/angiotensin receptor blockers, 62.62% vs. 38.16% (p<0.001); and a combination of all four medications, 39.32% vs. 14.47% (p<0.001). After adjusting baseline variables, the PFE system remained a significant contributor to adherence to these drugs use (P = 0.02).

**Conclusions:**

Reinforcement of patient education was associated with significant improvements in physicians’ adherence to guideline-recommended medical therapy after acute coronary syndrome.

## Introduction

Ischemic heart disease, especially acute coronary syndrome (ACS), is a leading cause of death worldwide[1]. However, because of the introduction of reperfusion therapy, intensive care, and medications for secondary prevention, the mortality rate of ACS has declined during the past 30 years. Nonetheless, studies have shown the suboptimal use of secondary preventive medications after discharge[2–5]. This nonadherence to the guidelines-recommended drug use is associated with worse patient outcomes[6]. Consequently, encouraging adherence to the guidelines is a relevant issue that affects the quality of care for those with ACS.

Measures have been proposed to enhance adherence to guidelines regarding ACS care. In previous literatures, the effects of a standardized order set, checkup list, reminder cards, and education regarding practice guidelines have been evaluated, resulting in variable degrees of improvement[7–9]. These quality-improvement tools were usually directed at physicians, who are responsible for medical decisions regarding ACS care. Nonetheless, the effects of these tools were criticized by physicians. In 2014, an observational study in conjunction with a nationwide registry was initiated at our hospital and this study aimed to evaluate the current practices and outcomes of ACS care. One year after the registry was created, an electronic-based patient and family education (PFE) system was systemically embedded in our hospital information system (HIS) for all patients. The prospectively collected ACS database provided us with an opportunity to evaluate the effects of PFE on the quality of care for ACS patients. Accordingly, we initiated a before-and-after analysis of the usage rates of guideline-recommended medications after ACS. In addition, the patterns and factors associated with prescription of guideline-recommended medications were examined.

## Materials and methods

### Study design and study cohorts

From February 2015 to April 2017, a prospective observational study in conjunction with a nationwide registry of ACS patients was performed to investigate ACS care at our hospital. Patients with an admission diagnosis of ST-segment elevation myocardial infarction (STEMI), non-ST segment elevation myocardial infarction (NSTEMI), and unstable angina according to the International Classification of Diseases, Ninth Revision (ICD-9), were prospectively enrolled. During the study period, the PFE system, which is electronic-based, was implemented at our HIS on January 4, 2016. Other interventions or policy changes were not initiated during the study period. To evaluate the impact of the PFE system, a retrospective before-and-after analysis was performed based on our ACS registry database. Patients discharged after the PFE system was implemented were defined as the intervention group; patients discharged before it was implemented was defined as the control group.

### Ethics statement

The study was approved by the Institutional Review Board of the National Taiwan University Hospital, Hsin-Chu Branch. Informed consent for participation in the observation cohort was obtained from all participants in the prospective registry; however, the requirement for such consent was waived for the retrospective analysis.

### Data collection

Demographics, clinical characteristics, medications, biochemistry data and in-patient therapies were collected by a trained study coordinator. Data regarding medications used at admission, during the hospital stay, after discharge, and during regular follow-up were collected. To establish a complete lipid profile, we used the Friedewald formula to estimate low-density lipoprotein cholesterol levels if they had not been directly measured. Chronic kidney disease (CKD) was defined as an estimated glomerular filtration rate of <60 mL/min/1.73 m^2^ calculated using the modification of diet in renal disease formula.

### PFE system

Before the implementation of the PFE system, educations for care after discharge were usually described to patients or their family members by nurse practitioners at the time of discharge. However, educations might have been implemented for shorter periods than required or not implemented at all due to limitations of the patients or caregivers. The completeness of the educations was not audited, and the comprehension of patients was not evaluated. The content of educations was not formatted; therefore, the information was dependent on the nurse practitioners who provided it. There was usually not enough time for feedback or questions from patients.

**Fig 1:**
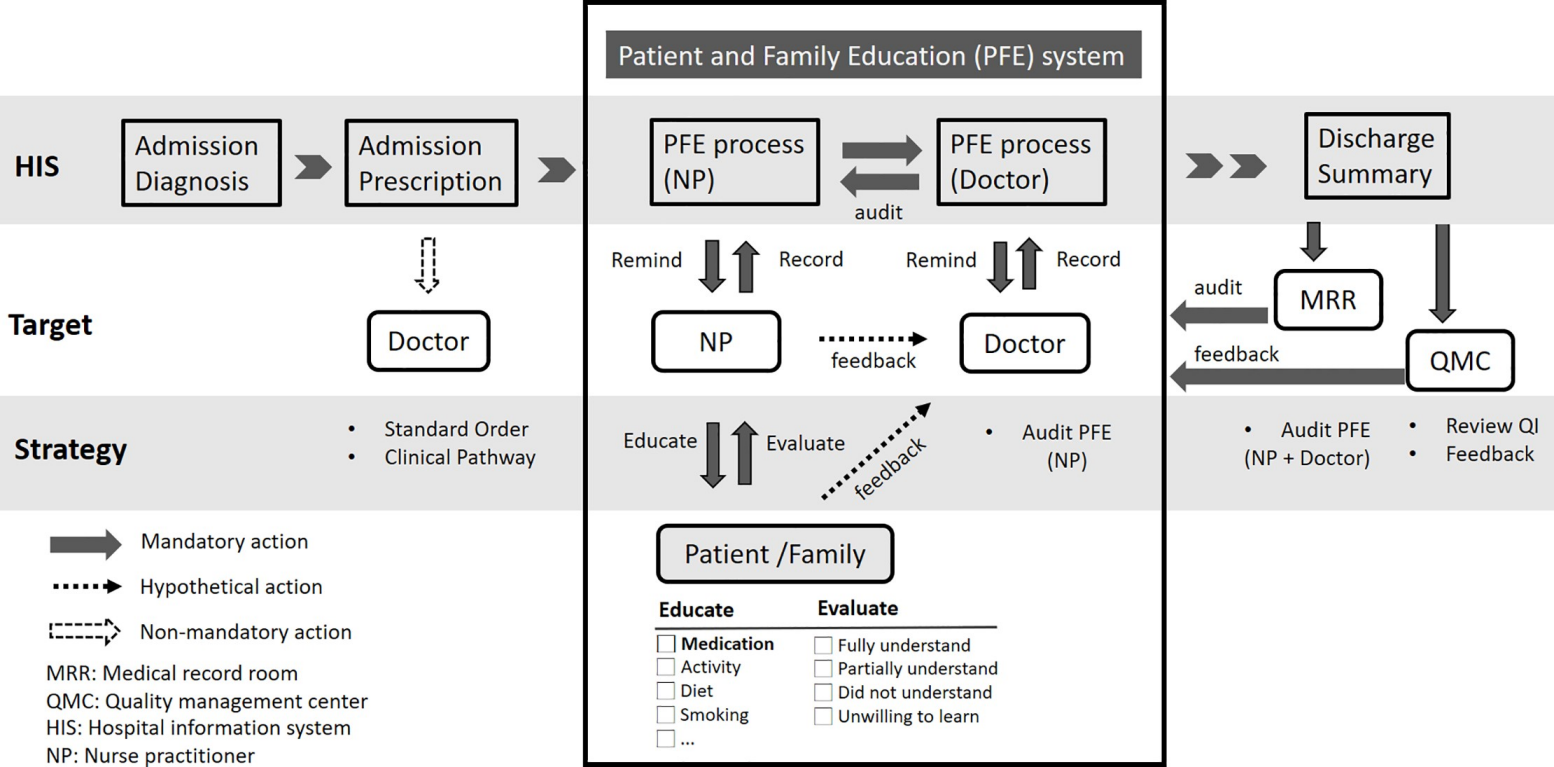
Patient and family education records displayed on a separate screen and linked with an icon on the main patient summary screen. Four topics were recorded: the target of education, clinical diagnosis, content of education, and evaluation of the effectiveness.

**Fig 2:**
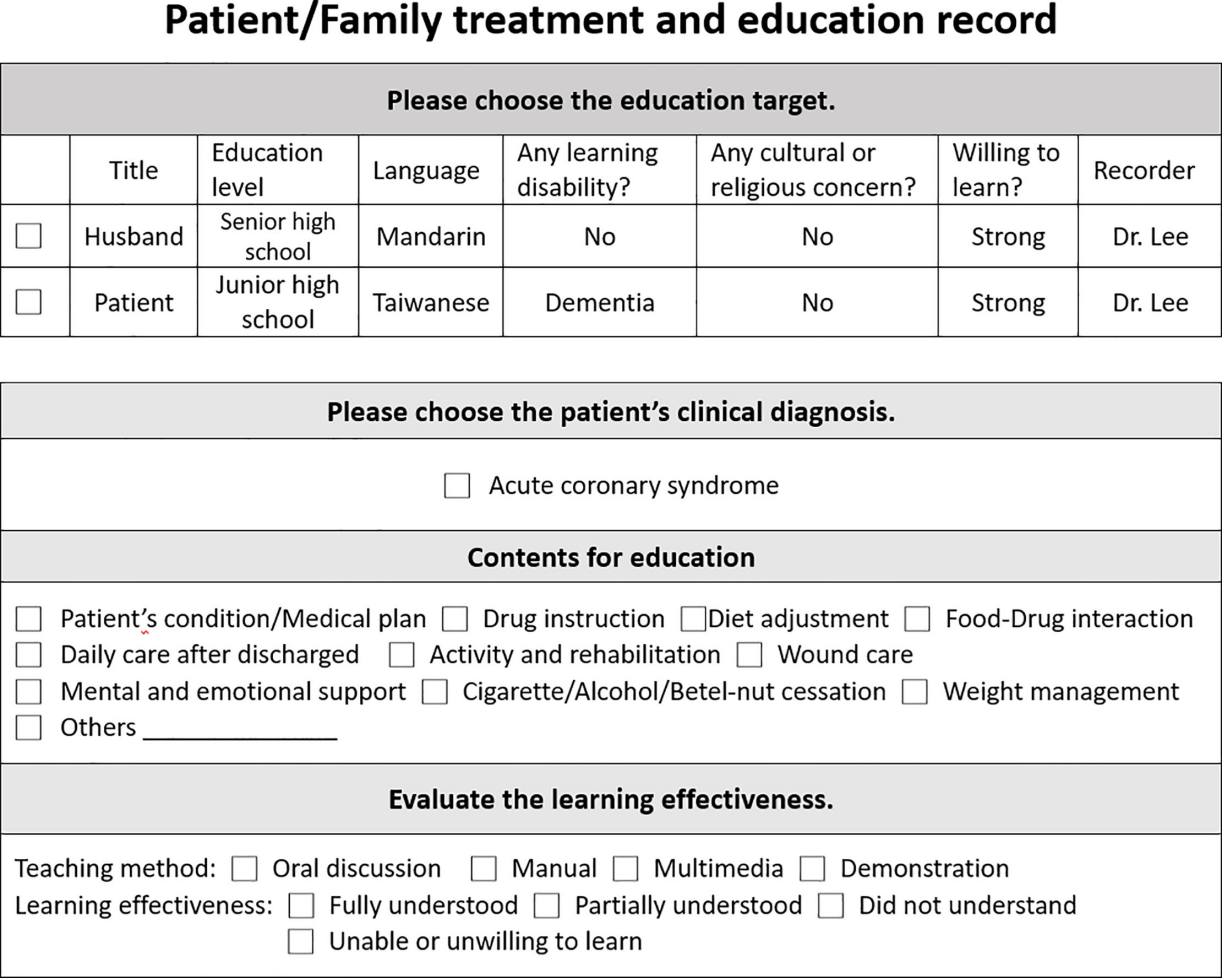
Illustration of the process of the patient and family education (PFE) system in the hospital information system (HIS) and the roles of caregivers during the PFE process. MMR, medical record room; NP, nurse practitioner; QI, quality improvement; QMC, quality management center.

The PFE system was designed to reinforce patient (or family member) education during hospitalization. It was embedded in our HIS and linked to an icon within the main patient summary screen. After the patient was admitted and the diagnosis was input in the system, the PFE icon was linked to a separate PFE window of the HIS. The PFE window consisted of four components: target of education, topic of education, content of education, and evaluation of effectiveness (**Fig 1**). For ACS patients, the education contents included symptoms, risk factors, diet, exercise, smoking cessation, and evidenced-based medications. A formatted introduction stating the purpose, directions, interactions, and adverse effects of guideline-recommended medications was provided for drug education. The patient’s understanding of this information was evaluated and documented. All education activities listed in the PFE window were required to be completed and confirmed by the nurse practitioners. Then, the completed PFE window was transferred to the attending physician; this window appeared in the to-do list. The physician was obligated to review and countersign the PFE window before the medical records were passed to the medical records department. When the patient was discharged, the information in the PFE window was audited by the staff in the medical records department (**Fig 2**).

### Statistical analysis

Data are presented as means (standard deviation [SD]) for normally distributed variables and median (interquartile range [IQR]) for non-normally distributed variables. Comparisons of continuous variables were performed using the Student t-test or Mann-Whitney U test as appropriate, and comparisons of proportions were performed using the chi-square test. Univariate and multivariable logistic regression analyses were used to identify possible predictors of adherence to the use of all appropriate drugs. All analyses were performed using SAS statistical software (SAS System for Windows, version 9.4; SAS Institute, Cary, NC, USA). All reported p values were two-sided, and the significance level was set at *p*<0.05.

## Results

### Characteristics of the study population

A total of 282 patients with ACS were enrolled in the study. Table 1 shows the demographic and clinical characteristics of the study population. There were 30.5% STEMI and 69.5% NSTEMI and unstable angina patients. The registry had a strong gender bias; two-thirds of the patients were male. The mean age of the patients was 68.5 years (SD, 14.2). Regarding the risk factors, 41.9% had dyslipidemia, 46.6% had diabetes, 76.0% had hypertension, 25.5% had chronic kidney disease, 23.0% had a family history of premature cardiovascular disease, and 26.3% were current smokers. Furthermore, 63.1% patients underwent reperfusion therapy, 57.8% underwent percutaneous coronary intervention (PCI), and 5.32% underwent a coronary artery bypass graft (CABG) procedure. Most of these patients (89.4%) were cared for by physicians of the cardiology specialty.

**Table 1:** Characteristics and clinical details of patients with acute coronary syndrome.

Variable^†^	All patients (n = 282)	PFE	Control	*p*-value^‡^
		n = 206	n = 76	
**Demographics**				
Sex, n (%)				0.850
Male	188 (66.67)	138 (66.99)	50 (65.79)	
Female	94 (33.33)	68 (33.01)	26 (34.21)	
Age (year), n (%)	68.46±14.17	67.13±14.56	72.05±12.42	0.009
<60	81 (28.72)	69 (33.50)	12 (15.79)	0.014
60–74	92 (32.62)	63 (30.58)	29 (38.16)	
≥75	109 (38.65)	74 (35.92)	35 (46.05)	
Height (cm)	162.75±8.80	162.79±8.89	162.66±8.57	0.921
Weight (kg)	67.23±15.05	67.17±15.06	67.39±15.13	0.920
BMI (kg/m^2^), n (%)	25.36±4.51	25.29±4.45	25.55±4.73	0.699
Underweight (<18.5)	13 (5.22)	10 (5.29)	3 (5.00)	1.000
Normal (18.5–24.9)	113 (45.38)	86 (45.50)	27 (45.00)	
Overweight (25–29.9)	90 (36.14)	68 (35.98)	22 (36.67)	
Obese (≥30)	33 (13.25)	25 (13.23)	8 (13.33)	
Transferred from another hospital	92 (33.09)	68 (33.50)	24 (32.00)	0.814
Specialty of the attending physician				0.375
Cardiologist	252 (89.4%)	190 (90.5%)	62 (86.1%)	
Non-cardiologist	30 (10.6%)	20 (9.5%)	10 (13.9%)	
**Type of ACS**				
Type of ACS, n (%)				0.526
STEMI	86 (30.50)	65 (31.55)	21 (27.63)	
UA+NSTEMI	196 (69.50)	141 (68.45)	55 (72.37)	
**Risk factors**				
Family history, n (%)	55 (23.01)	53 (30.64)	2 (3.03)	<0.001
Current smoker, n (%)	66 (26.29)	52 (28.42)	14 (20.59)	0.211
CKD, n (%)	72 (25.53)	47 (22.82)	25 (32.89)	0.085
Dyslipidemia, n (%)	103 (41.87)	66 (37.29)	37 (53.62)	0.020
Diabetes, n (%)	130 (46.59)	88 (43.14)	42 (56.00)	0.056
Hypertension, n (%)	212 (75.99)	155 (75.98)	57 (76.00)	0.997
**Reperfusion therapy (in hospital)**				
PCI during admission, n (%)	163 (57.80)	128 (62.14)	35 (46.05)	0.015
CABG during admission, n (%)	15 (5.32)	5 (2.43)	10 (13.16)	0.001

ACS, acute coronary syndrome; BMI, body mass index; CABG, coronary artery bypass graft; CKD, chronic kidney disease; NSTEMI, non-ST segment elevation myocardial infarction; PCI, percutaneous coronary intervention; PFE, patient and family education; STEMI, ST-segment elevation myocardial infarction; UA, unstable angina.

†Values are the mean ± standard deviation or number (percentage).

‡Differences between groups were evaluated by the χ2 test and two sample t-test.

Seventy-six patients were enrolled before implementation of the PFE system (control group) and 202 patients were enrolled after it was implemented (intervention group). Baseline characteristics of the two groups are provided in Table 1. The two groups were comparable regarding most demographic and clinical characteristics, except for age (younger in the intervention group), family history of cardiovascular disease and dyslipidemia history (more in the intervention group), and reperfusion therapy during hospitalization (more PCI and fewer CABG procedures in the intervention group). There was no significant difference in the specialty of the attending physicians between the control and intervention periods.

### Prescription of guideline-recommended medications

Table 2 presents the rates of prescribing guideline-recommended medications at the time of discharge. For the whole cohort, the prescription rate was 70.9% for dual antiplatelet therapy (DAPT) agents, 63.8% for statins, 71.6% for beta-blockers, 58.9% for angiotensin-converting enzyme (ACE) inhibitors/angiotensin receptor blockers (ARBs), and 32.6% for all four medications. Significant improvements in prescription usage rates were found after the PFE system was implemented, including DAPT agents (79.6% vs. 47.4%; *p*<0.001), statins (74.8% vs. 34.2%; *p*<0.001), beta-blockers (81.1% vs. 46.1%; *p*<0.001), and ACE inhibitors/ARBs (62.6% vs. 38.2%; *p* = 0.002), compared with before it was implemented. When adherence was determined by a combination of all four drugs, the prescription usage rate was higher after the PFE was implemented (39.3 vs. 14.5%; *p*<0.001).

**Table 2:** Prescriptions of guideline-recommended medicines for ACS stratified by groups before and after the audit program was implemented.

Variable	All patients (n = 282)	PFE	Control	*p*-value^†^
		n = 206	n = 76	
**Medication**				
Adherent to DAPT agents, n (%)	200 (70.92)	164 (79.61)	36 (47.37)	<0.001
Adherent to statins, n (%)	180 (63.83)	154 (74.76)	26 (34.21)	<0.001
Adherent to β-blockers, n (%)	202 (71.63)	167 (81.07)	35 (46.05)	<0.001
Adherent to ACE inhibitors/ARBs, n (%)	158 (56.03)	129 (62.62)	29 (38.16)	<0.001
**Combination**				
Adherent to all drugs, n (%)	92 (32.62)	81 (39.32)	11 (14.47)	<0.001

Values are number (percentage).

†Differences between groups were evaluated by the χ2 test and two-sample t-test.

ACE, angiotensin-converting enzyme; ACS, acute coronary syndrome; ARBs, angiotensin receptor blockers; DAPT, dual antiplatelet therapy; PFE, patient and family education.

DAPT: antiplatelet therapy combined with aspirin, Clopidogrel, or Ticagrelor; all drugs: DAPT+statin+β-blocker+ACE inhibitor/ARBs.

### Factors associated with the use of guideline-recommended medications

Prescriptions of DAPT agents were associated with a family history of premature cardiovascular disease, diabetes, PCI during the index admission, and the PFE system (Table 3). Prescriptions of statins were associated with STEMI, CKD, dyslipidemia, PCI, and the PFE system. Prescriptions of beta-blockers were associated with family history, hypertension, PCI, and the PFE system. Prescriptions of ACE inhibitors/ARBs were associated with family history, CKD, hypertension, PCI, and the PFE system. Adherence to using all respective drugs was associated with smoking habits, STEMI, PCI, and the PFE system.

**Table 3:** Factors associated with exposure to optimal preventive medications.

Variable^†^	DAPT (n = 282)			Statin (n = 282)			β-Blocker (n = 282)			ACEi/ARB (n = 282)			All respective drugs (n = 282)		
	Adherent (n = 200)	Nonadherent (n = 82)	*p*-value^‡^	Adherent (n = 180)	Nonadherent (n = 102)	*p*- value^‡^	Adherent (n = 202)	Nonadherent (n = 80)	*p*- value^‡^	Adherent (n = 158)	Nonadherent (n = 124)	*p*- value^‡^	Adherent (n = 92)	Nonadherent (n = 190)	*p*- value^‡^
**Sex, n (%)**			0.078			0.599			0.304			0.107			0.243
Male	127 (63.50)	61 (74.39)		118 (65.56)	70 (68.63)		131 (64.85)	57 (71.25)		99 (62.66)	89 (71.77)		57 (61.96)	131 (68.95)	
Female	73 (36.50)	21 (25.61)		62 (34.44)	32 (31.37)		71 (35.15)	23 (28.75)		59 (37.34)	35 (28.23)		35 (38.04)	59 (31.05)	
**Age (year)**	69.12±14.40	66.85±13.53	0.224	67.49±14.36	70.16±13.72	0.130	67.56±14.34	70.73±13.55	0.091	68.16±14.08	68.84±14.33	0.690	67.28±15.00	69.03±13.75	0.333
<60, n (%)	55 (27.50)	26 (31.71)	0.592	57 (31.67)	24 (23.53)	0.308	61 (30.20)	20 (25.00)	0.087	47 (29.75)	34 (27.42)	0.602	29 (31.52)	52 (27.37)	0.724
60–74, n (%)	64 (32.00)	28 (34.15)		58 (32.22)	34 (33.33)		71 (35.15)	21 (26.25)		54 (34.18)	38 (30.65)		30 (32.61)	62 (32.63)	
≥75, n (%)	81 (40.50)	28 (34.15)		65 (36.11)	44 (43.14)		70 (34.65)	39 (48.75)		57 (36.08)	52 (41.94)		33 (35.87)	76 (40.00)	
**Current smoker, n (%)**	47 (26.40)	19 (26.03)	0.951	45 (28.48)	21 (22.58)	0.305	49 (28.16)	17 (22.08)	0.313	36 (26.28)	30 (26.32)	0.995	27 (34.62)	39 (22.54)	0.044
**Family history, n (%)**	48 (28.40)	7 (10.00)	0.002	40 (26.14)	15 (17.44)	0.125	48 (28.07)	7 (10.29)	0.003	38 (28.79)	17 (15.89)	0.019	24 (30.00)	31 (19.50)	0.069
**Type of ACS, n (%)**			0.254			0.014			0.330			0.463			0.006
STEMI	65 (32.50)	21 (25.61)		64 (35.56)	22 (21.57)		65 (32.18)	21 (26.25)		51 (32.28)	35 (28.23)		38 (41.30)	48 (25.26)	
UA+NSTEMI	135 (67.50)	61 (74.39)		116 (64.44)	80 (78.43)		137 (67.82)	65 (32.18)		107 (67.72)	89 (71.77)		54 (58.70)	142 (74.74)	
**CKD**	53 (26.50)	19 (23.17)	0.560	37 (20.56)	35 (34.31)	0.011	50 (24.75)	22 (27.50)	0.633	32 (20.25)	40 (32.26)	0.022	17 (18.48)	55 (28.95)	0.059
**Dyslipidemia**	79 (44.89)	24 (34.29)	0.128	53 (32.92)	50 (58.82)	<0.001	71 (40.11)	32 (46.38)	0.371	60 (43.17)	43 (40.19)	0.639	28 (33.73)	75 (46.01)	0.065
**Diabetes, n (%)**	102 (51.00)	28 (35.44)	0.019	83 (46.63)	47 (46.53)	0.988	100 (49.75)	30 (38.46)	0.090	75 (47.47)	55 (45.45)	0.738	36 (39.13)	94 (50.27)	0.080
**Hypertension, n (%)**	153 (76.50)	59 (74.68)	0.749	135 (75.84)	77 (76.24)	0.941	161 (80.10)	51 (65.38)	0.010	130 (82.28)	82 (67.77)	0.005	73 (79.35)	139 (74.33)	0.357
**PCI**	142 (71.00)	21 (25.61)	<0.001	129 (71.67)	34 (33.33)	<0.001	133 (65.84)	30 (37.50)	<0.001	114 (72.15)	49 (39.52)	<0.001	73 (79.35)	90 (47.37)	<0.001
**CABG**	9 (4.50)	6 (7.32)	0.338	8 (4.44)	7 (6.86)	0.385	11 (5.45)	4 (5.00)	1.000	7 (4.43)	8 (6.45)	0.453	2 (2.17)	13 (6.84)	0.155
**PFE implementation**	164 (82.00)	42 (51.22)	<0.001	154 (85.56)	52 (50.98)	<0.001	167 (82.67)	39 (48.75)	<0.001	129 (81.65)	77 (62.10)	0.002	81 (88.04)	125 (65.79)	<0.001

†Values are the mean ± standard deviation or number (percentage).

‡Differences between groups were evaluated by the χ2 test and two sample t-test.

ACEi, angiotensin-converting enzyme inhibitor; ACS, acute coronary syndrome; ARB, angiotensin receptor blocker; CABG, coronary artery bypass graft; CKD, chronic kidney disease; DAPT, dual antiplatelet therapy; NSTEMI, non-ST segment elevation myocardial infarction; PFE, patient and family education system; STEMI, ST-segment elevation myocardial infarction; UA, unstable angina.

### Univariate and multivariate logistic regression analyses of factors associated with the use of guideline-recommended medications

A logistic regression analysis was performed to evaluate the effects of relevant clinical factors on using guideline-recommended prescription medications (**Table 4**). The univariate analysis indicated that smoking habits, STEMI, PCI, and the audit program were significantly associated with adherence to the use of guideline-recommended medications. After adjustments were made for the multivariable model, only PCI and the audit program were associated with adherence to the use of guideline-recommended medications.

**Table 4:** Univariate and multivariate logistic regression analysis of factors associated with all respective drugs adherence (n = 282).

	Adherence to all drugs			
	Univariate analysis		Multivariate analysis	
Variable	OR (95% CI)	P-value	OR (95% CI)	P-value
**Sex**				
Male	1		1	
Female	0.733 (0.436–1.235)	0.244	0.552 (0.240–1.271)	0.163
**Age**	0.991 (0.974–1.009)	0.332	1.010 (0.982–1.040)	0.488
**Current smoker**				
No	1		1	
Yes	1.819 (1.011–3.272)	0.046	1.578 (0.662–3.761)	0.304
**Family history**				
No	1		1	
Yes	1.77 (0.953–3.285)	0.071	1.443 (0.617–3.376)	0.397
**ACS type**				
STEMI	2.082 (1.227–3.532)	0.007	1.711 (0.807–3.625)	0.161
NSTEMI+UA	1		1	
**CKD**				
No	1		1	
Yes	0.556 (0.301–1.027)	0.061	0.642 (0.251–1.644)	0.356
**Dyslipidemia**				
No	1		1	
Yes	1.674 (0.966–2.900)	0.066	1.560 (0.708–3.436)	0.270
**Diabetes**				
No	1		1	
Yes	0.636 (0.383–1.056)	0.081	0.551 (0.261–1.161)	0.117
**Hypertension**				
No	1		1	
Yes	1.327 (0.727–2.422)	0.357	2.089 (0.876–4.982)	0.097
**PCI during admission**				
No	1		1	
Yes	4.269 (2.391–7.621)	<0.001	3.572 (1.598–7.986)	0.002
**CABG during admission**				
Non-CABG	1		1	
CABG	0.303 (0.067–0.067)	0.121	0.781 (0.085–7.207)	0.828
**PFE implementation**				
No	1		1	
Yes	3.829 (1.906–7.691)	<0.001	3.034 (1.192–7.725)	0.020

ACEi, angiotensin-converting enzyme; ACS, acute coronary syndrome; ARB, angiotensin receptor blocker; CABG, coronary artery bypass graft; CKD, chronic kidney disease; F, female; M, male; NSTEMI, non-ST segment elevation myocardial infarction; PCI, percutaneous coronary intervention; PFE, patient and family education; STEMI, ST-segment elevation myocardial infarction; UA, unstable angina.

CKD: serum creatinine >1.7 for men and serum creatinine >1.5 for women. DAPT: aspirin and Clopidogrel or Ticagrelor. All respective drugs: DAPT+statin+β-blocker+ACEi/ARB.

## Discussion

A previous nationwide registry in Taiwan and recent international registries showed that the use of guideline-recommended medications was usually suboptimal for ACS patients[2–4]. Our single-center cohort study demonstrated similar findings at a regional hospital. We found that the factor most relevant to these results was PCI during hospitalization. Additionally, significant improvements in the use of all medications were noted after implementation of the PFE system at our hospital.

Our study provided a novel method that may be helpful for future initiatives regarding improvements in adherence to drugs prescription. Factors related to physicians, including lack of familiarity, lack of agreement, or lack of attention to guidelines, had been thought to be the most critical for guideline adherence, and several tools were developed to standardize the care provided by physicians so that it is evidence-based. However, the use and effects of these quality-improvement measures varied widely among studies. For example, the effects of electronic reminder systems for physicians have been controversial because their use and effectiveness are usually attenuated because they are a disruption to other work[10]. The effects of an audit and feedback also varied among studies that mostly targeted physicians[11]. Standard orders improved the utilization of evidenced-based guidelines for those with ACS[12]. However, considerable variations in the rates of the use of standard orders across hospitals were found[13]. Resistance to standard orders by physicians is common because they interfere with autonomy and they are not always appropriate for every patient. Therefore, these physician-targeted approaches are suboptimal in clinical practice.

The PFE system targets the whole health care system, including patient, to facilitate the implementation of education, including guideline recommendations. After a diagnosis of ACS is recorded in the HIS, a PFE icon is displayed on the main patient screen as a reminder. Then, patient education activities are initiated early after hospitalization rather than late before discharge. Early initiation of patient education increases the opportunities for interactions between patients and physicians. Moreover, formatted content for education-based guidelines is introduced to the patients, and the effectiveness of education is enhanced by evaluations and adjustments facilitated by the PFE system. Finally, the implementation of patient education is audited at different stages, both during hospitalization and after discharge. Multilevel auditing also reinforces the implementation of patient education.

Although the PFE system does not target physicians directly, adherence to guidelines regarding drugs prescription significantly improved. The most plausible reason may have been that multifaceted interactions occurred among patients, nurse practitioners, and attending physicians during the PFE process. Early and comprehensive patient education during hospitalization enhances communication between patients and physicians. After patient education, feedback and questions from patients may serve as reminders to physicians and reinforce guideline adherence. During the process of patient education, deviations from guidelines are noted by nurse practitioners and serve as feedback to the attending physicians. Medicine based on the physician’s memory or habits is not always reliable. Focusing as much on the patients as on the physician allows the opportunity to optimize guideline adherence. Our findings suggested that increasing interactions between patients and physicians may complementarily aid in reinforcing the physicians’ adherence.

Our study highlighted not only the impact of the PFE system but also the relevant adherence factors at a regional hospital. However, factors other than the PFE system should be addressed. Our study identified a clinical subgroup with great potential for improvement. Patients who did not undergo PCI were less likely to use guideline-recommended medications. Similar findings have been reported in previous studies[14–17]. Several explanations for this phenomenon were proposed. PCI might not be utilized due to multiple comorbidities or poor general conditions, such as peptic ulcer, gastrointestinal bleeding, renal insufficiency, chronic obstructive pulmonary disease, or advanced age. These factors might be limitations or contraindications to using secondary preventive drugs. However, the severity of ACS might not be serious, thus leading physicians to focus less attention on secondary prevention. Identifying this caveat might help to remind physicians to focus attention on non-PCI patients and to improve guideline adherence.

The prescription rate in our study was worse than that of randomized trials. However, it was similar to the real-world results, such as those of the Taiwan ACS registry and others, which were also substantially worse[5, 18]. Fortunately, most of our prescription rates after the PFE system was implemented were higher than those of a previous nationwide ACS registry (DAPT agents, 80% vs. 75%; statins, 75% vs. 61%; and beta-blockers, 81% vs. 53%; however, this was not true for ACE inhibitors/ARBs, 62% vs. 63%)[5]. Similar to previous studies, the prescription rates were lowest for beta-blockers and renin angiotensin system inhibitors. The differences in prescription rates could be explained by two theories. First, in the updated United States and European ACS guidelines, ACE inhibitors/ARBs received a class I recommendation only for ACS patients with heart failure, hypertension, or diabetes, and beta-blockers received a class I recommendation only for ACS patients with heart failure[19, 20]. Second, reasons for not prescribing medications were not documented in this study. However, the adherence rate should be better than the prescription rate after excluding patients with contraindications or adverse effects.

Some limitations should be addressed. First, this study was an observational cohort study. Although the implementation of the PFE system occurred at a specific time, cohorts hospitalized before it was implemented and the cohorts hospitalized after it was implemented might not be completely comparable. Second, some factors distributed unequally between the control and intervention groups may be confounding factors for the adherence to medications. The patients in the intervention group were younger and more receptive to the education. They are more likely to be motivated to be healthy and live a longer life, and are, therefore, more likely to adhere to the medications. Moreover, physicians may have preoccupied perception and may be more motivated to prescribe guidelines directed medication due to a greater number of patients with dyslipidemia, with revascularization (PCI and CABG), and with premature family history of CAD in the intervention group. Third, we did not examine the long-term adherence of patients and did not record the doses of the drugs after discharge. Fourth, this was a single-center study performed at a regional hospital with a relatively small sample size; therefore, the generalizability of our results might be limited to similar hospitals. Fifth, the detailed mechanisms for improvement after the PFE process were not clear. A prospective survey on the patients, nursing practitioners, and physicians is needed for evaluation of the mechanism. Finally, the sustainability of the improvement after discharge was not proven.

Our results suggested that the implementation of the PFE system was associated with a significant improvement in the use of guideline-recommended medications after ACS. However, to confirm the effects of the PFE system and investigate its mechanisms, randomized studies aimed at determining whether this strategy could optimize the quality of care must be conducted.

## Supporting information

S1 File What’s New? **1.** Our study demonstrates that implementation of electronic-based patient and family education system was associated with improvements in physicians’ adherence to guideline-recommended medications. **2.** Our results suggest that a quality-improving initiative focusing on the patients may help to change the behaviors of physicians.
(DOCX)
