# Supplementary material for: Reinforcement of patient education improved physicians’ adherence to guideline-recommended medical therapy after acute coronary syndrome
Source: PLoS One. 2019 Jun 6;14(6):e0217444. doi: 10.1371/journal.pone.0217444 (PMC6553689; doi:10.1371/journal.pone.0217444)
Supplement: S1 File — 1. Our study demonstrates that implementation of electronic-based patient and family education system was associated with improvements in physicians’ adherence to guideline-recommended medications. 2. Our results suggest that a quality-improving initiative focusing on the patients may help to change the behaviors of physicians. (DOCX) [file pone.0217444.s001.docx]

What’s New?

1. Our study demonstrates that implementation of electronic-based patient and family education system was associated with improvements in physicians’ adherence to guideline-recommended medications.

2. Our results suggest that a quality-improving initiative focusing on the patients may help to change the behaviors of physicians.
